# Biomechanical Vitreous Alterations Detected by Strain Elastography Beyond Conventional B-Mode Ultrasound in Psoriatic Disease

**DOI:** 10.3390/diagnostics16182924

**Published:** 2026-09-10

**Authors:** José Alexandre Mendonça, Marcel Alex Soares dos Santos, Alessandra Gambero

**Affiliations:** 1 Stricto Sensu Postgraduate Program in Health Sciences, Pontifical Catholic University of Campinas (PUC-Campinas), Campinas 13087-571, SP, Brazil; marceldossantos@yahoo.com.br (M.A.S.d.S.); alessandra.gambero@puc-campinas.edu.br (A.G.); 2 Ultrasonography/Rheumatology Service, School of Life Sciences, Pontifical Catholic University of Campinas (PUC-Campinas), Campinas 13087-571, SP, Brazil

**Keywords:** arthritis, psoriatic, ultrasonography, elasticity imaging techniques, optical coherence tomography, vitreous body, uveitis

## Abstract

Ocular involvement in psoriatic disease is often underrecognized, particularly when inflammation affects posterior segments and remains undetectable on routine examination or conventional imaging modalities. We report a 38-year-old woman with palmoplantar psoriasis and psoriatic arthritis, characterized by ultrasound-confirmed dactylitis and inflammatory left hip pain, receiving methotrexate and guselkumab. Disease activity was low (DAPSA 7.6; CRP 5.8 mg/L). She presented with increased intraocular pressure, progressive visual blurring, and headache of 8 months’ duration, without a previous history of uveitis. Multimodal imaging was performed, including slit-lamp examination, grayscale B-mode ocular ultrasound, optical coherence tomography (OCT), and strain elastography. Slit-lamp examination and grayscale B-mode ultrasound were unremarkable, with no detectable vitreous echoes (grade 0). OCT demonstrated preserved retinal architecture with subtle hyperreflective vitreous dots in the right eye, compatible with low-grade vitreous cellularity, while the left eye was unremarkable. In contrast, strain elastography revealed increased stiffness and marked heterogeneity within the vitreous and periocular tissues, suggesting biomechanical alterations not detected by conventional structural imaging. These findings suggest biomechanical alterations compatible with possible inflammatory tissue remodeling. In our patient, strain elastography revealed biomechanical heterogeneity that was not apparent on conventional B-mode ultrasound. These findings should be considered hypothesis-generating and warrant further investigation in prospective controlled studies.

**Figure 1:**
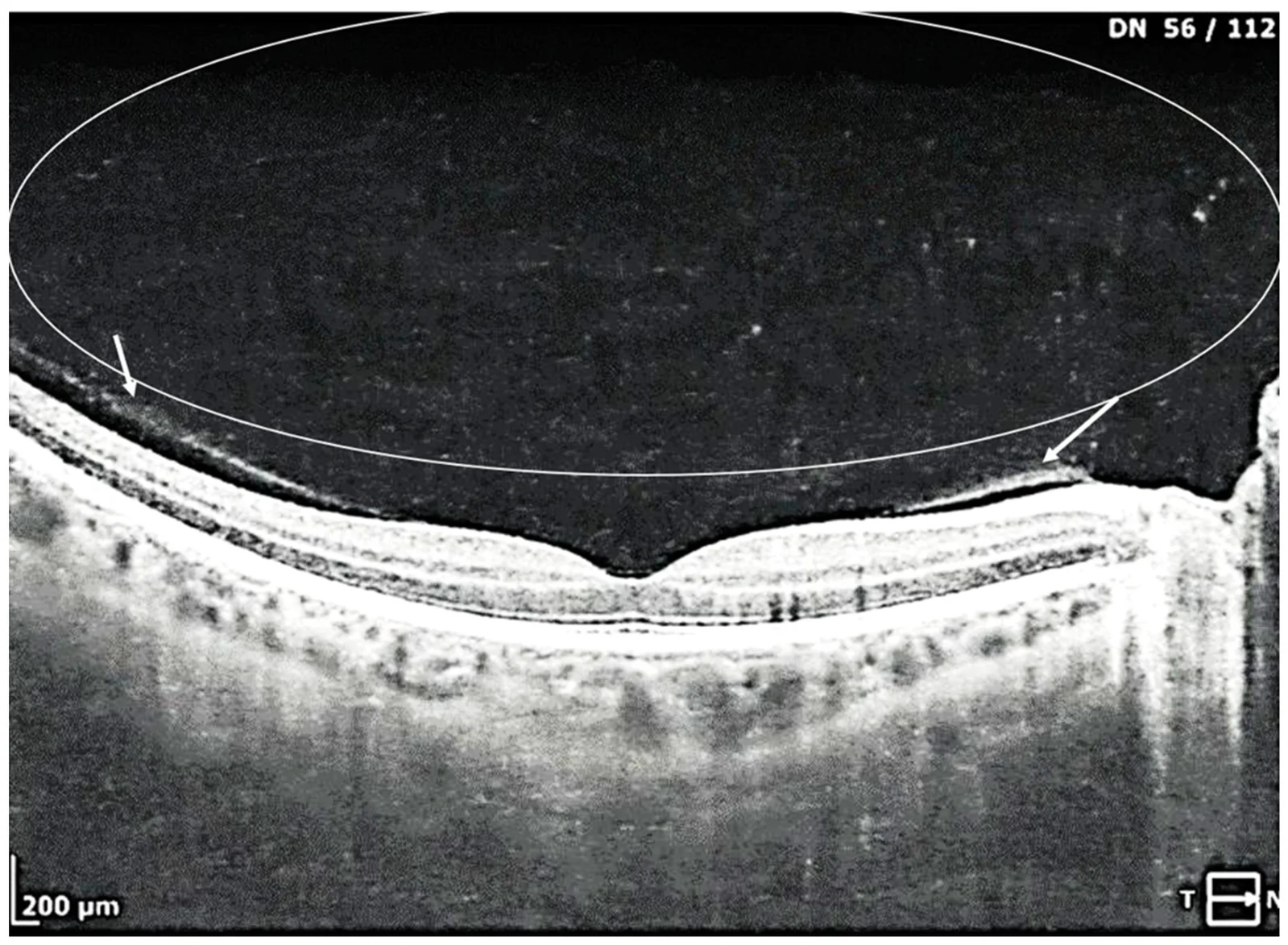
Macular SD-OCT (REVO, spectral-domain) demonstrating multiple hyperreflective punctate signals within the vitreous cavity (white arrows and circle), suggestive of mild vitreous cellularity. Previous studies have shown that OCT-derived vitreous signals may correlate with vitreous inflammatory activity and provide objective complementary information regarding intraocular inflammation. However, formal grading according to the SUN classification and the diagnosis of intermediate uveitis require comprehensive ophthalmologic clinical assessment and cannot be established based on OCT findings alone. Retinal architecture is preserved in both eyes, with intact foveal contour, preserved retinal layers from the internal limiting membrane to the retinal pigment epithelium, intact ellipsoid zone, and absence of disorganization of the retinal inner layers (DRIL). No intraretinal cysts, subretinal fluid, epiretinal membrane, vitreomacular traction, or macular edema are observed. The RPE–Bruch complex appears regular without signs of choroidal involvement [1,2,3].

**Figure 2:**
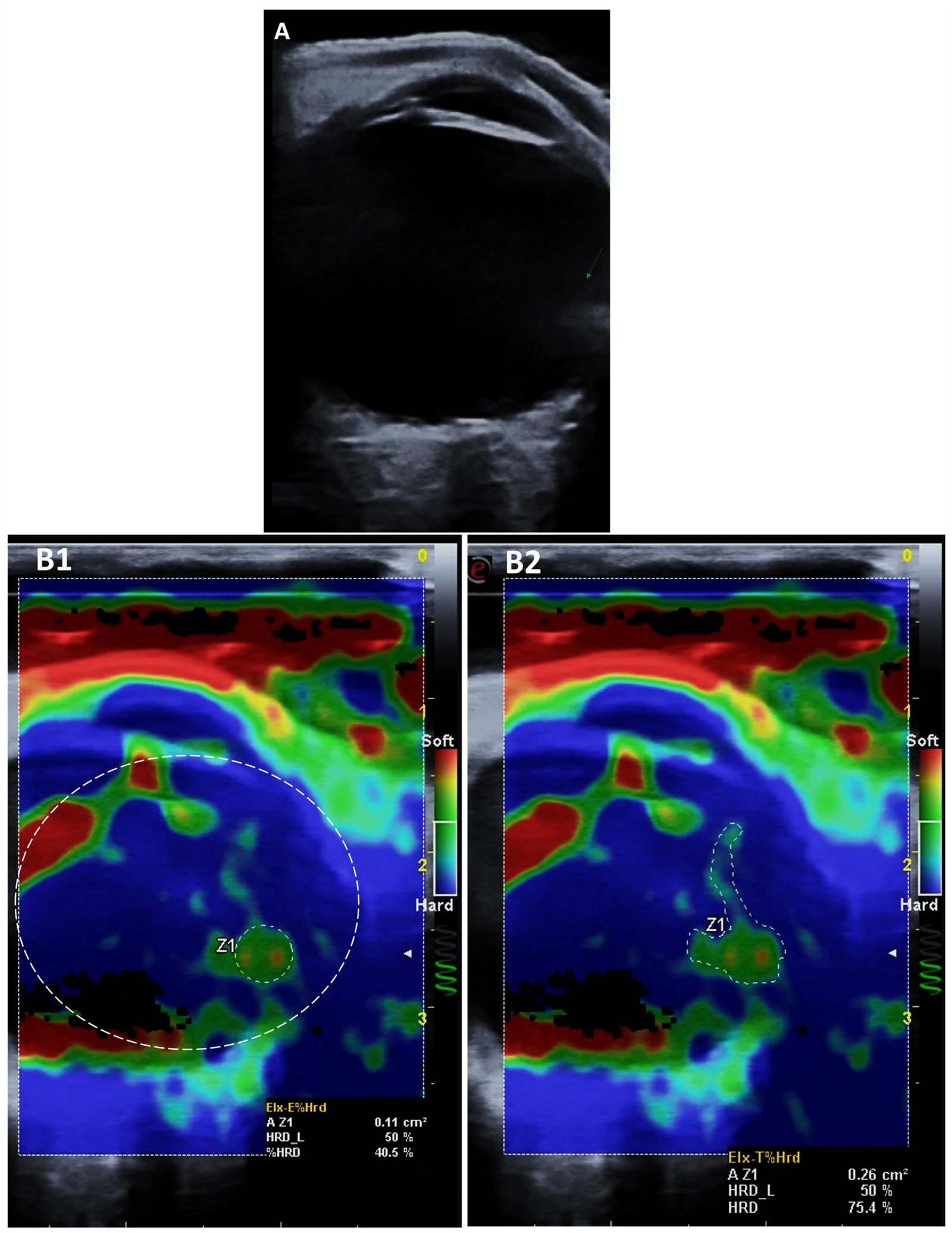
(**A**) High-frequency ocular ultrasound was performed using a MyLab™ Omega system (Esaote S.p.A., Genoa, Italy; eXP platform with ElaXto strain elastography software) equipped with a 6–19 MHz linear transducer. Mechanical and thermal indices were maintained within recommended ophthalmic safety limits. Gain settings were optimized during image acquisition to maximize the detection of low-amplitude vitreous echoes while maintaining adequate image quality and minimizing background noise. B-mode imaging demonstrates a uniformly anechoic vitreous cavity, corresponding to Grade 0 vitreous echoes. The longitudinal transpalpebral scan of the posterior segment shows no detectable hyperechoic dots, membranes, or particulate echoes. Vitreous echogenicity was semiquantitatively graded according to the distribution of hyperechoic dots within the vitreous cavity on B-mode ultrasonography: Grade 0 (anechoic vitreous), Grade 1 (≤25% of the vitreous area with echoes), Grade 2 (≥50%), and Grade 3 (≥75%) [4,5,6]. The present findings correspond to Grade 0, indicating the absence of detectable vitreous echoes according to this previously published ultrasonographic grading system [7]. This grading system characterizes vitreous echogenicity on B-mode ultrasonography, whereas the biological significance of these findings should be interpreted in conjunction with the overall clinical and multimodal imaging assessment. The arrow indicates a motion-related reverberation artifact at the sclera–retrobulbar interface, which should not be misinterpreted as a vitreous echo. (**B1**) Strain elastography of the vitreous demonstrates variation in relative stiffness according to the region of interest (ROI) sampling strategy. A wide dashed ellipse outlines the heterogeneous biomechanical pattern observed within the vitreous cavity. Within this environment, a small circular point-based ROI (Z1) yielded a relative stiffness value of 40.5%. (**B2**) A contour-based area ROI placed over the same anatomical region produced a higher value of 75.4%. These paired images illustrate how stiffness measurements obtained by strain elastography are dependent on ROI configuration and operator sampling when applied to heterogeneous vitreous tissue. The dashed ellipse indicates that biomechanical alterations are not confined to a single focal point but are distributed throughout the vitreous cavity. Two independent measurements were obtained from the abnormal vitreous region and compared with reference ocular structures, including the vitreous without apparent abnormalities, anterior chamber, retrobulbar fat, and optic nerve sheath. The operator performing elastographic acquisition and analysis was blinded to OCT findings. This figure depicts local biomechanical sampling within a globally heterogeneous vitreous environment, complementing grayscale ultrasound and OCT findings demonstrating vitreous structural and biomechanical heterogeneity. These images demonstrate localized biomechanical heterogeneity not apparent on conventional grayscale B-mode ultrasonography, potentially providing complementary biomechanical information alongside OCT findings. As a single-patient case report without controls, reproducibility assessment, or validated ocular strain elastography thresholds, these findings should be interpreted with caution. The relationship between biomechanical alterations and inflammatory activity remains uncertain; therefore, these observations should be considered exploratory and hypothesis-generating, warranting further validation in prospective controlled studies [8,9,10,11].

## Data Availability

The data presented in this study are available on request from the corresponding author due to privacy and ethical restrictions.

## References

[1] Qu Y., He Y., Saidi A., Xin Y., Zhou Y., Zhu J., Ma T., Silverman R.H., Minckler D.S., Zhou Q. (2018). In vivo elasticity mapping of posterior ocular layers using acoustic radiation force optical coherence elastography. Investig. Ophthalmol. Vis. Sci..

[2] Mendonça J.A., Coelho V.F.N., Nogueira H.S., Biselli L.G., Nucci L.B. (2023). Assessment of uveitis and complications by ultrasound in comparison with optical coherence tomography in psoriatic arthritis using etanercept. J. Clin. Exp. Ophthalmol..

[3] Herbort C.P., Takeuchi M., Papasavvas I., Tugal-Tutkun I., Hedayatfar A., Usui Y., Ozdal P.C., Urzua C.A. (2023). Optical coherence tomography angiography (OCT-A) in uveitis: A literature review and a reassessment of its real role. Diagnostics.

[4] Liu X., Kale A.U., Ometto G., Montesano G., Sitch A.J., Capewell N., Radovanovic C., Bucknall N., Beare N.A.V., Moore D.J. (2022). OCT Assisted Quantification of Vitreous Inflammation in Uveitis. Transl. Vis. Sci. Technol..

[5] Rosenbaum J.T., Dick A.D. (2018). The eyes have it: A rheumatologist’s view of uveitis. Arthritis Rheumatol..

[6] Mendonça J.A., de Figueiredo Torres Caivano R., Casani Rech I., Siste de Almeida Aoki I., Chagas Cavuto C. (2023). B-scan ultrasound evaluation for uveitis in inflammatory arthropathies: A systematic review. Eur. J. Rheumatol..

[7] Mendonça J.A., Pedri L.E., Andrade F., Biselli L., Nucci L.B. (2023). POS0901 Can B-mode ocular ultrasound assess vitritis in patients with axial spondyloarthritis and psoriatic arthritis?. Ann. Rheum. Dis..

[8] Mendonça J.A., Pedri L.E., Andrade F.R., Biselli L.G., Nucci L.B. (2025). Ultrasound evaluation of uveitis in patients with psoriatic arthritis. Braz. J. Dev..

[9] Sigrist R.M.S., Liau J., Kaffas A.E., Chammas M.C., Willmann J.K. (2017). Ultrasound elastography: Review of techniques and clinical applications. Theranostics.

[10] Asal N., Sayan C.D., Gökçınar N.B., Şahan M.H., Doğan A., İnal M. (2019). Evaluation of the optic nerve using strain and shear-wave elastography in pre-eclampsia. Clin. Radiol..

[11] Zhang J., Fan F., Zhu L., Wang C., Chen X., Gu X., Zhu J. (2022). Elasticity measurements of ocular anterior and posterior segments using optical coherence elastography. Opt. Express.

